# Long-Term Ketogenic Diet Induces Metabolic Acidosis, Anemia, and Oxidative Stress in Healthy Wistar Rats

**DOI:** 10.1155/2020/3642035

**Published:** 2020-06-19

**Authors:** Aryadi Arsyad, Irfan Idris, Andi A. Rasyid, Rezky A. Usman, Kiki R. Faradillah, Wa Ode U. Latif, Zidni I. Lubis, Aminuddin Aminuddin, Ika Yustisia, Yulia Y. Djabir

**Affiliations:** ^1^Department of Physiology, Faculty of Medicine, Hasanuddin University, Makassar, Indonesia; ^2^Biomedical Science Study Program, Postgraduate School, Hasanuddin University, Makassar, Indonesia; ^3^Department of Physiotherapy, Faculty of Health Science, University of Muhammadiyah Malang, Malang, Indonesia; ^4^Department of Nutrition, Faculty of Medicine, Hasanuddin University, Makassar, Indonesia; ^5^Department of Biochemistry, Faculty of Medicine, Hasanuddin University, Makassar, Indonesia; ^6^Laboratory of Clinical Pharmacy, Faculty of Pharmacy, Hasanuddin University, Makassar, Indonesia

## Abstract

**Background:**

Ketogenic diet has been used as supportive therapy in a range of conditions including epilepsy, diabetes mellitus, and cancer.

**Objective:**

This study aimed to investigate the effects of long-term consumption of ketogenic diet on blood gas, hematological profiles, organ functions, and superoxide dismutase level in a rat model.

**Materials and Methods:**

Fifteen male Wistar rats were divided into control (*n* = 8) and ketogenic (*n* = 7) groups. Controls received standard diet contained 52.20% of carbohydrates, 7.00% fat, and 15.25% protein; meanwhile, the ketogenic group received a high-fat-low-carbohydrate diet which contained 5.66% of carbohydrate, 86.19% fat, and 8.15% protein. All rats were caged individually and received 30g of either standard or high-fat-low-carbohydrate pellets. The experiment was carried out for 60 days before the blood samples were taken and analyzed to obtain blood gas, cell counts, organ biomarkers, and plasma antioxidant superoxide dismutase (SOD) levels.

**Results:**

The rats subjected to ketogenic diet experienced a marked decrease in body weight, blood sugar, and increased blood ketones (*p* < 0.05). The average blood pH was 7.36 ± 0.02 and base excess was −5.57 ± 2.39 mOsm/L, which were significantly lower than controls (*p* < 0.05). Hematological analysis showed significantly lower erythrocyte, hemoglobin, and hematocrit levels. No significant changes were found in alanine aminotransferase, aspartate aminotransferase, urea, and creatinine levels, indicating normal liver and kidney functions. Nevertheless, plasma SOD level significantly reduced with ketogenic diet.

**Conclusion:**

Long-term ketogenic diet induces metabolic acidosis, anemia, and reduced antioxidant enzyme level in rats following 60 days of consuming high-fat-low-carbohydrate diet.

## 1. Introduction

The ketogenic diet is a food regimen which consists of a high concentration of fat, with moderate/low protein and very low carbohydrate content. This type of diet triggers high production of ketone bodies derived from the breakdown of fat to produce energy [1]. Some studies show that the ketogenic diet has therapeutic benefits in a range of illnesses. It has been recommended as a supplementary therapy for polycystic ovary syndrome, acne, cancer, and respiratory distress [2]. It is also beneficial as anticonvulsant therapy to reduce the frequency of seizures in people with epilepsy [3, 4]. Ketogenic diet may also help to reduce HbA1C levels in people with type 2 diabetes, maintaining mood stability for people with bipolar disorder, and reducing cholesterol levels in obese patients [5].

A clinical study has demonstrated that a short-term ketogenic diet for 14 days might increase the concentration of ketone bodies in the blood, but it also improved the antioxidant capacity of the blood that contributes to reduced oxidative stress [6]. Another clinical trial has shown that consuming ketogenic diet for 20 days significantly reduced carbon dioxide deposits in the body, which may find clinical benefit in patients with increased PaCO_2_ due to respiratory failure [2].

Despite its popular use, some concerns arise on how ketogenic diet will affect the whole-body system. Since ketogenic diet replaces glucose with fat as the main source of energy, the body is forced to activate a series of fat metabolic processes to acquire energy [7]. Fat metabolic processes form acetyl coenzyme A (acetyl-CoA) as the main product, which then enters the citric acid cycle and is oxidized to produce ATP [8]. Acetyl-CoA that exceeds the availability of oxaloacetate and/or the activity of the citric acid cycle leads to an increase in ketone bodies (acetoacetate, *β*-hydroxybutyrate, and acetone). This process is called ketogenesis [9]. The ketone bodies formed from ketogenic diets are acidic; therefore, excessive excretion of these acids through kidneys may cause a decrease in alkaline reserves or bicarbonate ions (HCO_3_^−^) [10]. As a result, the implication of ketogenic diet reduced blood pH, leading to ketoacidosis [11].

Several animal models have been used to learn about the effect of a high-fat diet on the function of vital organs, such as the kidneys and liver [12, 13]. High-fat diet is more likely to trigger a reduction in mitochondrial quinone pool and is associated with increased mitochondrial reactive oxygen species (ROS) formation in the rat liver [14]. A high-fat diet has been shown to induce alteration in renal lipid metabolism in mice, especially the balance between lipogenesis and lipolysis, leading to the accumulation of lipid in the kidneys and, consequently, renal dysfunction [15].

To obtain more comprehensive data on how ketogenic diet may affect the whole-body system, this present study aimed to investigate the effects of long-term consumption of ketogenic diet on blood gas profiles, hematological parameters, organ functions, and antioxidant level in a rat model.

## 2. Materials and Methods

### 2.1. Preparation of Standard and Ketogenic Diet

Standard food was obtained from a manufacturer as standard pellets for rodents (AD2®, Indonesia), while the ketogenic food was prepared in our laboratory by involving a nutritionist. The ketogenic pellets contain 30% of nonpure fat mixed with 70% of goat fat (Table 1), which is formulated based on NutriSurvey® software to calculate the calorie intake and the percentage of macro and micronutrients per gram pellet. All ingredients were liquefied and mixed using a hand mixer and then frozen for 24 hours with the temperature of −20°C. The solidified material was then pulverized and molded into pellets. The standard and ketogenic pellets were then examined for their fat, protein, and carbohydrate contents at the Laboratory of Animal Food Chemistry, Faculty of Animal Science, Universitas Hasanuddin.

### 2.2. Experimental Protocols

Male Wistar rats weighing 200–330 g age 3–4 months (*n* = 15) were acclimated for 7 days in the laboratory before starting the experiment. At this stage, all rats received standard pellets and water *ad libitum*. Rats were cared for according to the standard for laboratory animal care, and all animal protocols have been approved by the Animal Ethics Committee of the Faculty of Medicine, Universitas Hasanuddin. Rats were divided into two groups. The first group (*n* = 8) received a standard diet, while the second group received the ketogenic diet for 60 days. This 60-day period of adult rat life is equivalent to ∼4 years of human life [17]. Each rat was caged individually and offered 30 g of food per day *ad libitum* and not subjected to calorie restriction. The remaining food was weighed every morning to record the calorie intake of each rat. The blood samples were withdrawn following 60 days of treatments and prepared for further analysis.

### 2.3. Analysis of Blood Gas, Hematological Parameters, Organ Biomarkers, and Superoxide Dismutase Level

The blood gas analysis was performed on rat whole blood immediately following blood sampling with the use of the i-Stat® analyzer (Abbott®). For hematological analysis, blood samples were collected using a BD® vacutainer with EDTA, centrifuged for 20 min with the rate of 3000 rpm before analyzed using a hematology analyzer (Thermo Scientific®). The organ biomarkers, such as alanine aminotransferase (ALT), aspartate aminotransferase (AST), creatinine, and urea were measured using Humalyzer 3500 (Human Global Diagnostic®) according to the instruction on the reagent kits (Human®). To measure plasma superoxide dismutase (SOD) level, the plasma was prepared based on instruction in Rat SOD for ELISA kit (Abbexa®) and analyzed with the enzyme-linked immunosorbent assay (ELISA) reader (Thermo Scientific®).

### 2.4. Analysis of Lipid Peroxidation Activity in Liver and Renal Tissues

At the end of the experiment, rats were anesthetized, euthanized, and laparotomy was performed. The liver and the kidneys of the rats were removed and immediately immersed in liquid nitrogen. Organs were weighed 400 mg and homogenized before adding 2 mL of phosphate buffer solution pH 7.4. The mixture is centrifuged at 3000 rpm for 20 minutes. The supernatant (0.5 mL) was mixed with 1 mL of 1% thiobarbituric acid and 1 mL of 1% trichloroacetic acid and heated to 100°C for 20 minutes. The mixture was then centrifuged at 3000 rpm for 10 minutes to separate the residue. Organ lipid peroxidation was measured as malondialdehyde (MDA) level (*λ* = 530 nm) using a UV-VIS spectrophotometer (Agilent®).

### 2.5. Statistical Analysis

The data obtained were analyzed using the SPSS IBM 23 software. Data distribution was examined using Kolmogorov–Smirnov to determine whether the data were normally distributed or not. The data that were normally distributed were subsequently analyzed using an independent *t*-test, while data that were not normally distributed were analyzed using the Mann–Whitney *U* test. A significant difference was achieved if *p* < 0.05 or very significant difference if *p* < 0.01. All data were presented in mean ± SEM.

## 3. Results

### 3.1. Long-Term Ketogenic Diet on Rats Causes Significant Weight Loss, Reduced Blood Glucose, and Increased Blood Ketone Levels

The food composition of the ketogenic pellet has far less carbohydrate (5.66% vs 52.20%) and much higher fat content (86.19% vs 7.00%) compared to the standard diet (Table 2). The calorie of the standard chow is 5.85 kCal/g, while that of the ketogenic pellet is 8.29 kCal/g. The average of daily calorie intake per rat in each week is depicted in Table 3. It is found that the standard group consumed more amount of food than the ketogenic group; hence, the calorie intakes of both groups are quite similar despite the difference in calories per gram food.

The difference in the diet composition was found to significantly affect the body weight, blood glucose, and blood ketone levels in the male rats after 60-day intake.  Table 4 shows the impact of ketogenic diet on rat body weight after 60 days. While all rats fed with standard diet gained weight after 2 months (on average ∼25% increase from baseline weight), the ketogenic-fed rats experienced a weight loss by around 100 g from their baseline body weight (∼40% loss).

**Figure 1:**
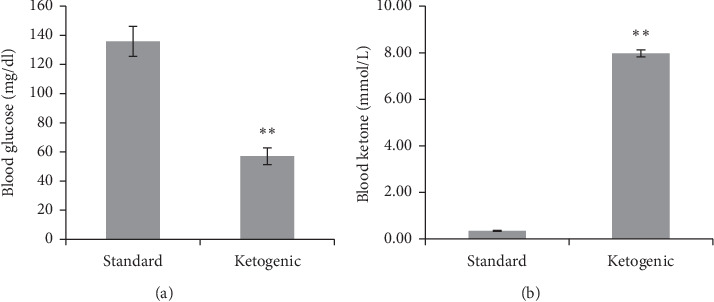
The level of blood glucose and blood ketone of rats consuming standard and ketogenic diet for 60 days. The symbol ∗∗ implies a very significant difference (*p* < 0.01) between groups.

Apart from weight loss, the blood glucose level of ketogenic-fed rats was significantly lower compared to the standard diet group (Figure 1). At this stage, the value of blood glucose was 57 ± 5.69 mg/dl, suggesting a hypoglycemic condition of the ketogenic diet group. Meanwhile, the level of blood ketone markedly elevated in the ketogenic group, about 8 times higher than the standard rats (7.97 ± 0.15 vs 0.34 ± 0.02 mmol/L).

### 3.2. Long-Term Ketogenic Diet Significantly Lowered Blood pH and Reduced Base Excess Level

The analysis of blood gas values demonstrates that the administration of the ketogenic diet for 60 days causes a significant alteration in blood gas homeostasis (Table 5). It was found there was a very significant decrease in blood pH of rats following 2 months of having a ketogenic diet compared to those fed with a standard diet (*p* < 0.01). The decrease in blood pH was not accompanied by significant changes in carbon dioxide pressure (pCO_2_), oxygen pressure (pO_2_), total carbon dioxide (TCO_2_), and hemoglobin oxygen saturation (SO_2_). Although the blood bicarbonate (HCO_3_^−^) level of the ketogenic group insignificantly decreased (19.74 ± 2.54 vs 22.75 ± 0.79 mmol/L), it was found that the group's base excess level was significantly lower compared to the standard group (*p* < 0.05).

### 3.3. Long-Term Ketogenic Diet Induces Anemia in Male Rats

The result of hematological analysis after receiving standard and ketogenic diets for 60 days is presented in Table 6. The ketogenic group appears to have slightly lower red blood cell (RBC) counts, significantly lower hemoglobin, and hematocrit, as well as significantly smaller mean corpuscular volume (MCV) and mean corpuscular hemoglobin (MCH) indices. These hematological abnormalities indicate that rats fed with the ketogenic diet were anemic.

### 3.4. Long-Term Ketogenic Diet Does Not Significantly Alter the Functions of Liver and Kidney

**Figure 2:**
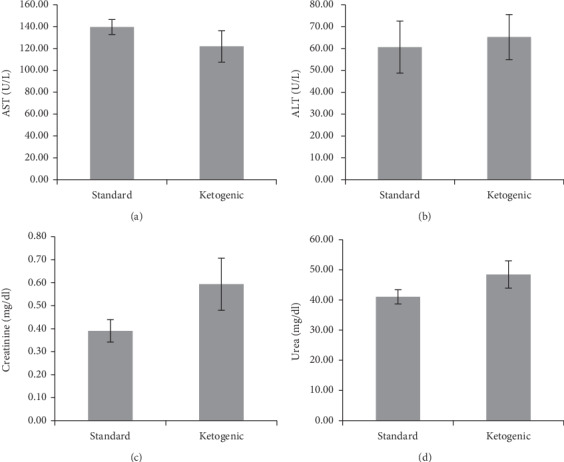
The level of aspartate aminotransferase (a), alanine aminotransferase (b), plasma creatinine (c), and plasma urea (d) in rats consuming standard and ketogenic diet for 60 days.

This study also measured the effect of a long-term ketogenic diet in rats on liver and renal functions. The result is presented in Figure 2. From the data, it is revealed that the levels of liver biomarkers, the alanine aminotransferase (ALT) and aspartate aminotransferase (AST), were not significantly different between the standard and ketogenic groups. However, when comparing the renal function test, a slight increase in plasma creatinine and urea levels was found in the ketogenic group compared to standard, although the difference was not statistically significant.

### 3.5. Long-Term Ketogenic Diet Increases Lipid Peroxidation and Reduces the Antioxidant Level

**Figure 3:**
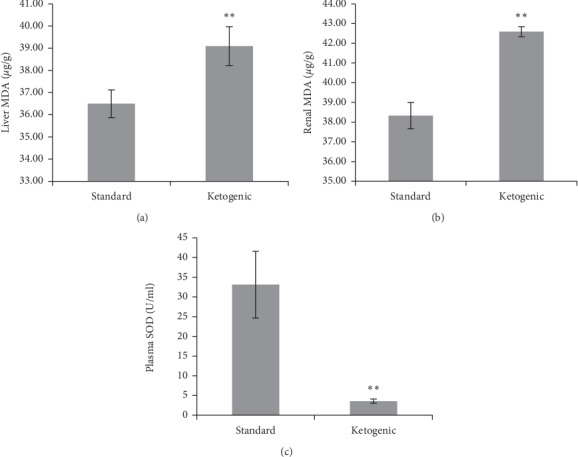
The level of liver malondialdehyde (a), renal malondialdehyde (b), and plasma superoxide dismutase (c) in standard and ketogenic-fed rats. The symbol ∗∗ implies a very significant difference (*p* < 0.01) between groups.

The level of lipid peroxidation and antioxidant activity could be a good indicator to reveal oxidative stress level in the system. In this study, it was found that the ketogenic diet in rats for 60 days may induce an increase in malondialdehyde (MDA) level in the liver and kidney (Figure 3). The increase of MDA level in both vital organs was very significant in the ketogenic group compared to standard (*p* < 0.01). The increase of MDA level in the ketogenic group was accompanied by a reduced level of antioxidant superoxide dismutase (SOD), which was ∼80% lower compared to that of standard (*p* < 0.01).

## 4. Discussion

The ketogenic diet has gained public attention since it is first introduced as an alternative therapy for pharmacoresistant epilepsy [18]. Nowadays, the use of ketogenic diet has expanded beyond epileptic therapy; indeed, its use in healthy individuals has become more popular, especially to those who wish to lose weight. Unfortunately, the benefits of ketogenic diet may come with side effects. This study examined the long-term effects of ketogenic diet in a healthy male rat model to obtain more information about the potential complications of this type of diet.

Standard food with its high carbohydrate content allows the body to use glucose as the main source of energy. When carbohydrate intake is more than sufficient to meet the needs of ATP, the body will physiologically convert glucose into glycogen as energy stores in tissues. Consumption of a diet rich in carbohydrates will also cause an increase in the amount of fat deposited in adipose tissue under the skin or in the abdominal cavity. This is the main reason for the increase in body weight of rats fed with a standard diet.

On the other hand, rats treated with the ketogenic diet had a significant weight loss as a result of induced ketosis. Ketogenic diet with high fat, low protein, and low carbohydrate composition renders the body depends on the process of gluconeogenesis, the formation of noncarbohydrate glucose, to produce energy [19]. When the fatty acids (fat content) are mainly used to produce energy, it will induce the formation of ketone bodies, such as acetoacetate, beta-hydroxybutyrate, and acetone. The presence of ketosis in the ketogenic group was confirmed by a significantly higher level of blood ketone (∼8 mmol/L) and a significantly low blood sugar level (<60 mg/dl). Apart from weight loss, the rats also experience a decrease in pH or acidosis, which occurs as a result of increased blood ketone level [11]. The ketone bodies are acidic; thus, an increase in ketone bodies in circulation may induce acidosis [11, 20].

Anemia is not uncommon side effects of high-fat diets. This study also found reduced hematological indices, such as RBC, hemoglobin, hematocrit, MCV, and MCH in rats fed with ketogenic diet for 60 days. Studies on epileptic children have revealed that ketogenic diet is more likely to cause anemia, which may occur due to dietary restriction, leading to copper deficiency [21, 22]. However, this complication of the ketogenic diet can be managed with copper supplementation.

In this study, the administration of ketogenic diet in rats for 60 days did not significantly alter liver and kidney function. Nevertheless, the plasma creatinine and urea of the ketogenic-fed rats were somewhat higher than standard-fed rats, which may suggest a minor effect of the ketogenic diet on renal function. This effect could be more striking if the duration of the ketogenic diet administration is prolonged.

It is interesting that although the liver and renal function were not significantly altered, the lipid peroxidation activity in both organs significantly increased. This was indicated by a significantly higher MDA level of liver and renal tissues in ketogenic-fed rats compared to those with a standard diet. Increased activity of lipid peroxidation could be triggered by the elevation of reactive oxygen species (ROS) in the organs and incapability of the antioxidant enzyme activity to protect cell membranes from ROS-induced damage. This result could emerge as a potential threat to both organs should the diet be sustained longer than the period investigated. In line with this, the plasma concentration of superoxide dismutase (SOD) significantly reduced in ketogenic-fed animals (*p* < 0.01), suggesting the presence of oxidative stress induced by long-term ketogenic diet in rats.

The reason why a ketogenic diet may induce oxidative stress has been explained in several studies. Ketone bodies are known to stimulate the mitochondria to produce more ATP compared to glucose [23–25]. However, fat metabolism requires more complex processes, such as reduction, oxidation, hydroxylation, and conjugation, which may elevate the production of reactive oxygen species (ROS) in the liver cells [26, 27]. If the release of ROS is in balance with the body's antioxidant activities, the occurrence of oxidative stress can be prevented. In contrast, if ROS formation has exceeded antioxidant levels, the free radicals will attack macromolecules, such as, proteins, polysaccharides, DNA, and cell membranes that contain polyunsaturated fatty acids, leading to cellular damage [28]. This study shows an increase in liver and renal MDA levels, which is accompanied by a decrease in plasma SOD after 60-day consumption of ketogenic diet. This might implicate a precaution on the long-term use of the ketogenic diet.

## 5. Conclusions

Despite the weight loss, low blood sugar, and high blood ketone, sustainable consumption of keto diet for 60 days in rats also instigated some concerning effects such as metabolic acidosis, anemia, and decreasing plasma antioxidant enzyme level. It is interesting that albeit a significant increase in lipid peroxidation activity on the liver and kidney, both organ functions were remained intact, at least during the period investigated.

## Figures and Tables

**Table 1:** Composition of standard and ketogenic diet.

Composition	Percentage
Standard diet^a^	
Water	12
Protein	15
Palm oil	7
Fiber	6
Calcium	7
Phosphor	0.7
Enzyme	0.1
Corns	52.2

Ketogenic diet^b^	
Water	—
Avocado	5.69
Chicken egg yolk	19.45
Roasted peanuts	4.86
Goat fat	70

^a^Formula is obtained from the commercial rodent chow label. ^b^Formula is prepared based on ketogenic diet for rats, with the ratio of 8.6 : 1 portion of fat:(carbohydrate + protein) [16].

**Table 2:** The comparison of carbohydrate, fat, and protein contents of standard and ketogenic diets obtained from food analysis.

Type of diet	Carbohydrate (%)	Fat (%)	Protein (%)
Standard	52.20	7.00	15.25
Ketogenic	5.66	86.19	8.15

**Table 3:** The average of daily calorie intake per rat each week in standard and ketogenic diet groups.

Diet	The calorie intake (kCal/day)							
	Week I	Week II	Week III	Week IV	Week V	Week VI	Week VII	Week VIII
Standard	83.11	81.24	81.13	82.83	80.62	78.29	76.19	75.57
Ketogenic	88.77	84.78	82.86	83.45	86.70	84.78	88.77	98.24

**Table 4:** Changes in rat body weight after receiving standard and ketogenic diets for 60 days.

Type of diet	*N*	Body weight	Mean ± SEM (g)	*p* value
Standard	8	Baseline	252 ± 20.61	0.001^*∗*^
		Posttreatment	319 ± 19.35	

Ketogenic	7	Baseline	260 ± 12.60	0.01^*∗*^
		Posttreatment	157 ± 06.40	

**Table 5:** The comparison of blood gas profiles of rats receiving standard and ketogenic diets for 60 days.

Blood gas	*N*	Diet	Mean ± SEM	*p* value
pH	8	Standard	7.52 ± 0.01	0.001^*∗*^
	7	Ketogenic	7.36 ± 0.02	

pCO_2_ (mmHg)	8	Standard	27.63 ± 1.34	1.00
	7	Ketogenic	35.72 ± 5.96	

pO_2_ (mmHg)	8	Standard	107.75 ± 2.93	0.32
	7	Ketogenic	88.14 ± 12.14	

HCO_3_^−^ (mmol/l)	8	Standard	22.75 ± 0.79	0.48
	7	Ketogenic	19.74 ± 2.54	

Base excess (mmol/l)	8	Standard	1.08 ± 0.43	0.04^*∗*^
	7	Ketogenic	0.32 ± 0.11	

TCO_2_ (mmol/l)	8	Standard	23.63 ± 0.84	0.56
	7	Ketogenic	20.86 ± 2.77	

SO_2_ (%)	8	Standard	98.63 ± 0.18	0.20
	7	Ketogenic	90.29 ± 6.18	

**Table 6:** The comparison of hematology profiles of rats receiving standard and ketogenic diets for 60 days.

Hematology parameters	*N*	Diet	Mean ± SEM	*p* value
RBC (10^6^/*μ*L)	8	Standard	8.04 ± 0.24	0.33
	7	Ketogenic	7.65 ± 0.29	

Hemoglobin (g/dl)	8	Standard	13.76 ± 0.33	0.02^*∗*^
	7	Ketogenic	11.98 ± 0.54	

Hematocrit (%)	8	Standard	39.90 ± 0.97	0.001^*∗*^
	7	Ketogenic	32.77 ± 1.69	

MCV (fL)	8	Standard	49.78 ± 1.40	0.001^*∗∗*^
	7	Ketogenic	42.67 ± 0.89	

MCH (pg)	8	Standard	17.15 ± 0.37	0.01^*∗*^
	7	Ketogenic	15.62 ± 0.19	

## Data Availability

The data used to support the findings of this study are available from the corresponding author upon request.
